# Transport of Photonic Bloch Wave in Arrayed Two-Level Atoms

**DOI:** 10.1038/s41598-018-20023-x

**Published:** 2018-01-24

**Authors:** Chih-Chun Chang, Lee Lin, Guang-Yin Chen

**Affiliations:** Department of Physics, National Chung Hsing University, Taichung, 402 Taiwan

## Abstract

In a quantum system of arrayed two-level atoms interacting with light, the interacted (dressed) photon is propagating in a periodic medium and its eigenstate ought to be of Bloch type with lattice symmetry. As the energy of photon is around the spacing between the two atomic energy levels, the photon will be absorbed and is not in the propagating mode but the attenuated mode. Therefore an energy gap exists in the dispersion relation of the photonic Bloch wave of dressed photon in addition to the nonlinear behaviors due to atom-light interactions. There follows several interesting results which are distinct from those obtained through a linear dispersion relation of free photon. For example, slow light can exist, the density of state of dressed photon is non-Lorentzian and is very large around the energy gap; the Rabi oscillations become monotonically decreasing in some cases; and besides the superradiance occurs at long wavelengths, the spontaneous emission is also very strong near the energy gap because of the high density of state.

## Introduction

In the past 20 years, the quantum effects of matter-light interactions have attracted considerable interests in the area of physics and applied sciences. There are many experimental and theoretical works studying quantum interactions between light and (artificial) atoms^1,2^ to explore fundamental sciences and modern technologies in several fields including atomic systems^3,4^, semiconducting^5–11^, superconducting^12–16^, and other condensed-matter systems^17–19^. For example, many novel researches have been inspired by controlling atomic and/or photonic states through manipulating electronic states by emissions and absorptions of photons in quantum optics^20^, quantum computing^13,14,19^, quantum transport^21^, and optical lattices, etc.

Nevertheless, the atom-photon interactions of systems of one (artificial) or few (artificial) atoms have already been investigated^13,20,22–25^ comprehensively. And many later researches have been conducted to study systems of many atoms, especially, arrayed atoms interacting with photonic fields^19,26–28^. As has been pointed out by Bloch that the eigenfunction (Bloch wave function) of a particle propagating in a lattice is a product of a plane wave and a periodic function with lattice symmetry. And the eigenvalue of a Bloch wave function is usually different from that of its corresponding plane wave which is the eigenfunction in free space. Therefore, for photon with lattice momentum *k* propagating in a system of arrayed atoms, its eigenfunction would be of Bloch type with eigenenergy other than the usual *ck*’s; that is, the interacted photon in this case would have a nonlinear dispersion relation rather than a linear one of free photon. In addition, as the energy of photon is around the spacing Δ*E* between two atomic energy levels, photons will be absorbed, and thus they are in an attenuated mode with a complex lattice momentum. Therefore, there is no real lattice momentum *ħk* corresponding to the energy *ħω* around Δ*E* in the photonic dispersion relation, and this is an energy gap within which photons are absorbed and cannot propagate far. It can be expected that, for interacted photons with nonlinear dispersion relation including energy gap(s), their physical behaviors would be different from those of free photons with a linear dispersion relation.

Nowadays, more people have directed their attentions to the interactions between arrayed atoms and photons, which lead to distinct phenomena such as the slow light^29–31^ in atomic medium, and the cavity-like act of the atom array^25,26^. Therefore, based on results of our previous works^32,33^, it is our motivation to go beyond plane wave eigenfunction of free photon with a linear dispersion relation, and to study the influences of the photonic Bloch wave of dressed (interacted) photon with energy gap in the nonlinear dispersion relation to an arrayed two-level atoms. And we would report in this paper some physical quantities and physical behaviors which are quite different from those obtained with a linear photonic dispersion relation. For example, the density of state of photon would not be exactly Lorentzian; the Rabi-like oscillation and the spontaneous emission of a Dicke state^25,26,34^ would show monotonic decreases in addition to oscillations; and the spontaneous emission of a Dicke state also shows intensive radiance at certain medium wavelengths along with the superradiance^10,35–38^ occurring at long wavelengths.

## Results

### Model

**Figure 1:**
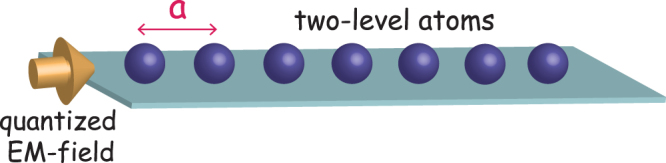
Model. *N* two-level atoms (*σ*_*i*_’s) on a linear lattice (*x*-direction) interacting with a quantized EM field $\overrightarrow{A}$ propagating along $\hat{x}$. The distance between adjacent atoms is defined as the lattice constant *a*.

Following our previous work^32^, we consider an array (*x*-direction) of *N* two-level atoms (*σ*_*i*_’s) with the distance between adjacent atoms *a* (lattice constant) interacting with a quantized EM field $\overrightarrow{A}$ through a quantum interaction that two-level atoms can be excited (de-excited) by absorbing (emitting) photons as is shown in Fig. 1. Assuming that the EM wave is propagating in the *x*-direction and uniform along $\hat{y}$, $\hat{z}$, we can then write the vector potential $\overrightarrow{A}$ as $\overrightarrow{A}(x)=\mathrm{(0},{A}_{y}(x),{A}_{z}(x))$, in the radiation gauge ($\nabla \cdot \overrightarrow{A}=0$). The Hamiltonian *H*_*em*_ for the EM field is

$${H}_{em}=\frac{1}{2}\,\int \,dx\,[{\dot{\overrightarrow{A}}}^{2}+{(\nabla \times \overrightarrow{A})}^{2}]=\int \,dx({\dot{A}}^{\dagger }\dot{A}+\nabla {A}^{\dagger }\cdot \nabla A),$$

where *field operators A*(*x*) & *A*^†^(*x*) are so defined $A=({A}_{y}+i{A}_{z})/\sqrt{2}$, ${A}^{\dagger }=({A}_{y}-i{A}_{z})/\sqrt{2}$ which describe annihilation and creation of one photon, respectively. To describe transitions between the ground state |*g*〉_*j*_ and the excited state |*e*〉_*j*_ of the two-level atom on the *j*th site, the raising and lowering operators are defined,

$${\sigma }_{j}^{+}\equiv |e{\rangle }_{j}{\langle g{|}_{j},{\sigma }_{j}^{-}\equiv |g\rangle }_{j}\langle e{|}_{j}.\,$$

In terms of the photonic field operators *A* & *A*^†^, and the raising and lowering operators for the two-level atoms, *σ*^+^ & *σ*^−^, the Hamiltonian of the whole system can be expressed as,

1 $$\begin{array}{rcl}H & = & {H}_{em}({A}^{\dagger },A)+{ {\mathcal H} }_{2LS}(\{{\sigma }_{z}\})+{ {\mathcal H} }_{int}(A,\{\sigma \}),\\ { {\mathcal H} }_{2LS}(\{{\sigma }_{z}\}) & = & \sum _{j}\,(\nu +i\delta )\frac{1+{\sigma }_{jz}}{2},\,{\sigma }_{jz}\equiv |e{\rangle }_{j}{\langle e{|}_{j}-|g\rangle }_{j}\langle g{|}_{j},\end{array}$$

2 $$\begin{array}{rcl}{ {\mathcal H} }_{int}(A,\{\sigma \}) & = & g\,\sum _{j}\,[{\sigma }_{j}^{+}A({x}_{j})+{A}^{\dagger }({x}_{j}){\sigma }_{j}^{-}],\end{array}$$

where ${ {\mathcal H} }_{2LS}(\{{\sigma }_{z}\})$ is the Hamiltonian of *N* two-level atoms with excitation energy *ν* of each atom (ground state energy is 0); and ${ {\mathcal H} }_{int}(A,\{\sigma \})$ is the Hamiltonian for the quantum interaction between atom and photon with the coupling constant $g\sim e\sim \sqrt{\mathrm{1/137}}$. In this paper, we use natural units by putting *ħ* = *c* = 1 as is widely adopted in field theory literatures, *e*.*g*., ref.^39^, p. 88. (Nevertheless, we shall put *ħ* & *c* back, if necessary.) Hence both the resonant angular frequency *ν* and the resonant energy *ħν* of the two-level atom are expressed as *ν* in the natural units.

Following the calculations in ref.^32^, the free propagator of the two-level atom is

3 $${{\rm{\Delta }}}_{j}^{\mathrm{(0)}}(t)=\int \,d\omega \,{\tilde{{\rm{\Delta }}}}^{\mathrm{(0)}}(\omega ){e}^{-i\omega t},\quad {\tilde{{\rm{\Delta }}}}^{\mathrm{(0)}}(\omega )=\frac{i}{\omega -\nu +i\delta }.$$

**Figure 2:**
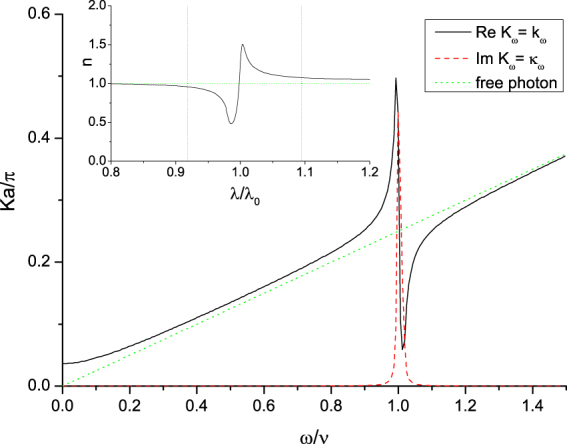
Dispersion. (**a**) Dispersion relations (the real part *k*_*ω*_*a*/*π* versus *ω*/*ν* [solid line], and the imaginary part *κ*_*ω*_*a*/*π* versus *ω*/*ν* [dashed line]) of photonic field propagating (outgoingly) in an array of two-level atoms. Energy gap exists and it is where *κ*_*ω*_ > 0. The dotted line is the dispersion relation of free photon. Here we choose *N* = 100, *g*^2^/*a* = 0.01, *ν* = 0.25*π*, and $\delta =3\ast {10}^{-3}\nu$. The *y*-axis is in unit of *ka*/*π*, and the *x*-axis is in unit of *ω*/*ν*; both of them are dimensionless. (**b**) The index of refraction *n* is shown in the inset. The *x*-axis is *λ*/*λ*_0_, with *λ*_0_ the wavelength at the resonant angular frequency *ν*. The two dotted lines show where the energy gap appears between 0.918*λ*_0_ & 1.094*λ*_0_.

The Green’s function of the EM field *G*(*x*, *t*; *x*′, *t*′) satisfies the Dyson’s equation as,

$$G(x,t;x^{\prime} ,t^{\prime} )={G}_{0}(x,t;x^{\prime} ,t^{\prime} )+{g}^{2}\,\sum _{j}\,G(x,t;{x}_{j},t^{\prime\prime} ){{\rm{\Delta }}}_{j}(t^{\prime\prime} -t^{\prime} ){G}_{0}({x}_{j},t^{\prime\prime} ;x^{\prime} ,t^{\prime} ),$$

or can be expressed in the following way in the momentum space,

4 $$\begin{array}{rcl}\tilde{G}{(k,\omega ;k^{\prime} ,\omega ^{\prime} )}^{-1} & = & \tilde{G}{(k^{\prime} +h,\omega ;k^{\prime} ,\omega )}^{-1}\delta (\omega ^{\prime} -\omega ){\delta }_{k^{\prime} +h,k}\\  & = & [{\tilde{G}}_{0}{(k,\omega )}^{-1}{\delta }_{k^{\prime} ,k}+2i\,{\rm{\Pi }}(\omega ){\delta }_{k^{\prime} +h,k}]\cdot \delta (\omega ^{\prime} -\omega ),\end{array}$$

where ${\tilde{G}}_{0}(k,\omega )=i/({\omega }^{2}-{k}^{2}+i\epsilon )$ is the free propagator of the EM field ($\epsilon \to {0}^{+}$), *h*’s the reciprocal lattice vectors (*h* = 2*nπ*/*a*, *a* the lattice constant), and Π(*ω*) is

5 $${\rm{\Pi }}(\omega )=-\frac{i{g}^{2}}{2a}{\tilde{{\rm{\Delta }}}}^{\mathrm{(0)}}(\omega ),$$

which represents the modification to the propagator (*self*-*energy*) of the EM wave due to atom-photon interaction. And it contains both real part and imaginary part which is originated from *δ*. Then the dispersion relation of photons can be obtained by solving the wave equation in momentum space, or, equivalently, solving the pole of the Green’s function in field theoretical treatment^32,40^,

6 $${a}^{2}{\rm{\Pi }}(\omega )\frac{\sin \,\omega a}{\omega a}+\,\cos \,\,\omega a-\,\cos \,\,ka=\mathrm{0,}$$

from which *ω*_*k*_ = *ω*(*k*) (or, *k*_*ω*_ = *k*(*ω*)) can be obtained; and *k*_*ω*_ vs. *ω* is depicted in Fig. 2. In fact, the eigenstate |Ψ_*k*_〉 corresponding to the eigenenergy (excitation spectrum) *ω*_*k*_ is a photonic Bloch state^33^. In Fig. 2, for 0.914*ν* < *ω* < 1.089*ν*, there is no real *k* which can satisfy the dispersion relation *ω*(*k*) = *ω*. Therefore, an energy gap appears in the range between 0.914*ν* & 1.089*ν*. Within this range, photons will be absorbed. During the revision of this paper, we were informed that there is an interesting similarity between our dispersion relation Eq. (6) and that of the system of surface plasmon polaritons^41^. For example, in ref.^41^, there is also a gap in their dispersion relation between the plasmon frequency and the surface plasmon frequency. It needs further explorations to study the connections between these two systems of light-matter interactions.

The index of refraction *n*(*ω*) can then be obtained^42^ (putting *c* back),

7 $$n(\omega )=\frac{c}{\omega }{\rm{Re}}\sqrt{{[{\rm{Re}}({k}_{\omega })]}^{2}-{[{\rm{Im}}({k}_{\omega })]}^{2}+2i\,{\rm{Re}}({k}_{\omega })\,{\rm{Im}}({k}_{\omega })},$$

and is depicted in the inset of Fig. 2. The function Π(*ω*) (Eqs (3) and (5)) plays an important role in the behavior of the index of refraction *n*(*ω*). By its definition, Π(*ω*) is proportional to the Green’s function of the two-level atoms ${\tilde{{\rm{\Delta }}}}^{\mathrm{(0)}}(\omega )$ which is the response of the two-level atoms to the impulse from other field, the photonic field in this case. Therefore, by the principle of action and reaction, Π(*ω*) represents the impact on the photonic field from the two-level atoms as is shown in the photonic dispersion relation *ω*_*k*_ (Eq. (6)). Were there no Π(*ω*), *ω*_*k*_ would become linear as the dispersion relation of the free photon. And it can be seen that ${\rm{\Pi }}(\omega )\,(=\frac{{g}^{2}\mathrm{/2}a}{\omega -\nu +i\delta })$ undergoes rapid and significant changes near the resonant angular frequency *ν*. We define $\bar{\lambda }(\omega )=2\pi c/\omega$ to be the wavelength of the free photon. As a result of significant changes of Π(*ω*) near the resonant angular frequency, the wavelength of the photonic Bloch wave *λ*(*ω*) varies from a value less than $\bar{\lambda }(\omega )$ to a value greater than it. Consequently, the index of refraction $n(\omega )=\mathop{\lambda }\limits^{-}(\omega )/\lambda (\omega )$ becomes less than unity as *ω* > *ν*, or the corresponding wavelength *λ* < *λ*_0_, with *λ*_0_ the wavelength at the resonant angular frequency *ν* (Fig. 2). This phenomenon also appears in other single-resonance media^43^. The figure of the index of refraction *n* vs. *λ*/*λ*_0_(=*ν*/*ω*) shown in the inset of Fig. 2 is qualitatively similar to the first part of Figure 5.6–4 of ref.^43^, p. 188.

Following similar calculations done in ref.^33^, the dressed photon propagator can be obtained as,

8 $$\begin{array}{rcl}\tilde{G}(l,\omega ;k,\omega ) & = & \langle l|\tilde{{\mathscr{U}}}(\omega )|k\rangle \\  & = & \sum _{l^{\prime} ,k^{\prime} }\,\langle l|{{\rm{\Psi }}}_{l^{\prime} }\rangle \langle {{\rm{\Psi }}}_{l^{\prime} }|\tilde{{\mathscr{U}}}(\omega )|{{\rm{\Psi }}}_{k^{\prime} }\rangle \langle {{\rm{\Psi }}}_{k^{\prime} }|k\rangle \\  & = & |F(k;{\omega }_{k}{)|}^{2}\frac{i}{{\omega }^{2}-{\omega }_{k}^{2}+i\epsilon }{\bar{\delta }}_{l,k},\end{array}$$

where $\tilde{{\mathscr{U}}}(\omega )$ is the Fourier transform of the time evolution operator ${\mathscr{U}}(t)$, ${\bar{\delta }}_{p,q}$ specifies crystal momentum conservation (*i*.*e*., ${\bar{\delta }}_{p,q}=1$, if *p* = *q* + 2*nπ*/*a*; ${\bar{\delta }}_{p,q}=0$, otherwise); and |Ψ_*k*_〉 is the photonic Bloch state,

9 $$\begin{array}{rcl}{{\rm{\Psi }}}_{k}(x) & = & {u}_{k}(x)\frac{{e}^{ikx}}{\sqrt{{\mathscr{N}}a}},\end{array}$$

10 $$\begin{array}{rcl}{\rm{with}}\,{u}_{k}(x) & = & 2\,{\rm{\Pi }}(\omega )F(k,{\omega }_{k})f(x;k,{\omega }_{k}),\,({u}_{k}(x+a)={u}_{k}(x)),\end{array}$$

11 $$\begin{array}{rcl}f(x;k,{\omega }_{k}) & = & -\frac{a}{2{\omega ^{\prime} }_{k}}\frac{{e}^{-ik(a-{\rm{\Delta }}x)}\,\sinh ({\omega ^{\prime} }_{k}{\rm{\Delta }}x)+{e}^{ika}\,\sinh \,[{\omega ^{\prime} }_{k}(a-{\rm{\Delta }}x)]}{\cos ({\omega ^{\prime} }_{k}a)-\,\cos (ka)},\end{array}$$

12 $$\begin{array}{rcl}F(k,{\omega }_{k}) & = & \langle k|{{\rm{\Psi }}}_{k}\rangle =\frac{1}{\mathrm{2|}{\rm{\Pi }}(\omega )|}{[\frac{1}{a}{\int }_{0}^{a}dx|f(x;k,{\omega }_{k}{)|}^{2}]}^{-\mathrm{1/2}},\end{array}$$

and Δ*x* = *x* − *a*[*x*/*a*] ([] is the Gauss notation), ${\omega ^{\prime} }_{k}=\sqrt{{\omega }_{k}^{2}+i\epsilon }$. Since |Ψ_*k*′_〉 and |Ψ_*k*′+2*nπ*/*a*_〉 are the same, without loss of generality, we can require the Bloch state indices *k*′ and *l*′ in the above equation (Eq. (8)) to be in the same Brillouin zone as *k* and *l*.

### Density of state

The dressed propagator of the two-level atom at the *i*th site Δ_*i*_(*t*;*t*′) = Δ_*i*_(*t*′ − *t*) satisfies the following Dyson’s equation^32^,

13 $${{\rm{\Delta }}}_{i}(t^{\prime} -t)={{\rm{\Delta }}}_{i}^{\mathrm{(0)}}(t^{\prime} -t)+{g}^{2}\,\int \,d{t}_{1}d{t}_{2}\,{{\rm{\Delta }}}_{i}(t^{\prime} -{t}_{2})\,G({x}_{i},{t}_{2};{x}_{i},{t}_{1})\,{{\rm{\Delta }}}_{i}^{\mathrm{(0)}}({t}_{1}-t\mathrm{).}$$

Please notice that by incorporating the renormalized photon propagator in Eq. (13), the renormalized propagator of the two-level atom Δ_*i*_(*t*' − *t*) includes all those amplitudes that photon is emitted at site *i* and repeatedly absorbed/emitted at other sites and finally absorbed at site *i*. The above Dyson’s equation can also be expressed in the following way in the momentum space,

14 $$\begin{array}{rcl}i\tilde{{\rm{\Delta }}}{(\omega )}^{-1} & = & i{\tilde{{\rm{\Delta }}}}^{\mathrm{(0)}}{(\omega )}^{-1}+i{g}^{2}\,\int \,\frac{dk}{2\pi }\tilde{G}(k,\omega )\\  & = & \omega -\nu +i\delta -{g}^{2}\,\int \,\frac{dk}{2\pi }\frac{|F(k;{\omega }_{k}{)|}^{2}}{{\omega }^{2}-{\omega }_{k}^{2}+i\varepsilon }\end{array}$$

15 $$\begin{array}{rcl} & = & \omega -\nu +i\delta -i\,{g}^{2}\frac{|F({k}_{\omega };\omega {)|}^{2}}{(-\mathrm{)2}\omega }(\frac{\partial {k}_{\omega }}{\partial \omega }),\end{array}$$

where the last term is self-energy, in the language of field theory, and it can be viewed as the renormalization correction *δν*(*ω*) from atom-photon interactions to the energy *ν*,

16 $$\nu (\omega )=\nu +\delta \nu (\omega ),\,\delta \nu (\omega )=-i\,{g}^{2}\frac{|F({k}_{\omega };\omega {)|}^{2}}{2\omega }(\frac{\partial {k}_{\omega }}{\partial \omega }).$$

Eq. (14) can also be written as,

17 $$i\tilde{{\rm{\Delta }}}{(\omega )}^{-1}=\omega -\nu +i\delta -{g}^{2}\,\int \,\frac{d{\omega }_{k}}{2\pi }{(\frac{\partial {\omega }_{k}}{\partial k})}^{-1}\frac{|F(k;{\omega }_{k}){|}^{2}}{{\omega }^{2}-{\omega }_{k}^{2}+i\varepsilon }.$$

**Figure 3:**
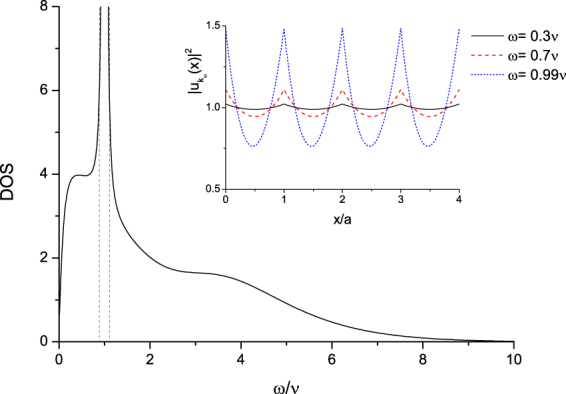
DOS. (**a**) Density of photonic Bloch state. The two dotted lines show where the energy gap appears between 0.914*ν* & 1.089*ν*. (**b**) The inset shows the probabilities of the Bloch wave functions $Na|\langle x|{{\rm{\Psi }}}_{{k}_{\omega }}\rangle {|}^{2}=|{u}_{{k}_{\omega }}(x{)|}^{2}$ at different *ω*’s: 0.3*ν* (solid), 0.7*ν* (dashed), & 0.99*ν* (dotted). Here, we use the same parameters as in Fig. 2. (In the natural units (*ħ* = *c* = 1) taken in this paper, the resonance energy is *ν*. And actually it is *ħν*, if we put *ħ* back).

By comparing the above equation with the conventionally adopted density of state^44^ *ρ*_*E*_(*ω*), and by Eq. (12), it can be identified that *ρ*_*E*_(*ω*)*dω* is

18 $${\rho }_{E}(\omega )d\omega =d\omega (\frac{\partial {k}_{\omega }}{\partial \omega })\cdot |F({k}_{\omega };\omega {)|}^{2}=d\omega (\frac{\partial {k}_{\omega }}{\partial \omega })\cdot |\langle {k}_{\omega }|{{\rm{\Psi }}}_{{k}_{\omega }}\rangle {|}^{2},$$

in which $d\omega (\frac{\partial {k}_{\omega }}{\partial \omega })$ is the number of plane wave state |*k*_*ω*_〉 between *ω* & *ω* + *dω*, and $|\langle {k}_{\omega }|{{\rm{\Psi }}}_{{k}_{\omega }}\rangle {|}^{2}$ the probability of overlapping between the plane wave state |*k*_*ω*_〉 and the corresponding photonic Bloch state $|{{\rm{\Psi }}}_{{k}_{\omega }}\rangle$. Thus *ρ*_*E*_(*ω*)*dω* is the number of (photonic) Bloch state $|{{\rm{\Psi }}}_{{k}_{\omega }}\rangle$ between *ω* & *ω* + *dω*, and *ρ*_*E*_(*ω*) is the density of state (DOS) which is depicted in Fig. 3. It is non-Lorentzian and is very large around the energy gap. A lot of physical behaviors of photon propagations, e.g. Rabi oscillations and Spontaneous emissions, depend on the DOS *ρ*_*E*_(*ω*). And we will show this later in the Discussion section.

It should be noted that as *ω* is within the energy gap (*k*_*ω*_ is complex), the state |*k*_*ω*_〉 is corresponding to an attenuated wave, and $\langle x|{{\rm{\Psi }}}_{{k}_{\omega }}\rangle$ is not a periodic Bloch wave. Therefore, it is beyond our previous discussions based on the (periodic) Bloch wave, and *ρ*_*E*_(*ω*) does not carry the meaning of density of periodic photonic Bloch state in the energy gap.

## Discussion

The Dicke state^34^ is defined as,

$$|+{\rangle }_{{k}_{0}}=\frac{1}{\sqrt{N}}\,\sum _{i}\,{e}^{i{k}_{0}{x}_{i}}|i\rangle \mathrm{.}$$

where, |*i*〉 = |*g*_1_, *g*_2_, …, *g*_*i*−1_, *e*_*i*_, *g*_*i*+1_, …, *g*_*N*_〉 is the state with the *i*th two-level atom being in its excited state (|*e*〉) and other atoms being in their ground states (|*g*〉). Since *k*_0_ appears in the form as ${e}^{\pm i{k}_{0}{x}_{i}}$ in this model, without loss of generality, we can restrict *k*_0_ to be within the 1st Brillouin zone.

### Rabi oscillations before renormalization

**Figure 4:**
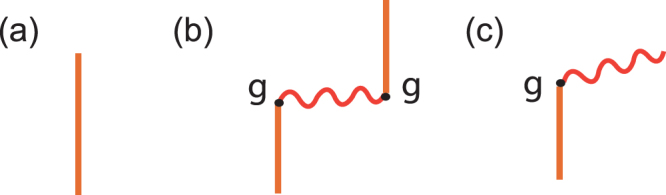
Feynman diagrams. (**a**) Feynman diagram of $\langle i|{\mathscr{U}}(t)|i\rangle$ which is the direct propagation of a two-level atom at site *i* to itself after a time evolution *t*. (**b**) Feynman diagram of $\langle \,j\ne i|{\mathscr{U}}(t)|i\rangle$ which is the indirect propagation of a two-level atom at site *i* to a different site *j* after a time evolution *t* through emitting and absorbing a photon. (**c**) Feynman diagram of $\langle \gamma ({x}_{j})|{\mathscr{U}}(t)|i\rangle$ which is the spontaneous emission of a two-level atom at site *i* emitting a photon to *j* after a time evolution *t*.

From Fig. 4(a,b), and by Eq. (8), with the time evolution operator ${\mathscr{U}}(t)$, the amplitude of an initial Dicke state remains unchanged after a time period *t* can then be expressed as

19 $$\begin{array}{rcl}{}_{{k}_{0}}\langle +|{\mathscr{U}}(t)|+\rangle _{{k}_{0}} & = & \frac{1}{N}\,\sum _{i,\,j}\,{e}^{i{k}_{0}({x}_{i}-{x}_{j})}\langle j|{\mathscr{U}}(t)|i\rangle \\  & = & \int \,\frac{d\omega }{2\pi }{e}^{-i\omega t}\tilde{{\rm{\Delta }}}(\omega )+\frac{{g}^{2}}{N}\,\sum _{i,j(\ne i)}\,{e}^{i{k}_{0}({x}_{i}-{x}_{j})}\\  &  & \cdot \int \,\frac{d\omega }{2\pi }\,\int \,\frac{dk}{2\pi }{e}^{-i\omega t}\tilde{{\rm{\Delta }}}{(\omega )}^{2}\,{e}^{ik({x}_{j}-{x}_{i})}\tilde{G}(k,\omega )\end{array}$$

20 $$\begin{array}{rcl} & = & \int \,\frac{d\omega }{2\pi }{e}^{-i\omega t}\tilde{{\rm{\Delta }}}(\omega )+\frac{{g}^{2}}{N}\,\int \,\frac{d\omega }{2\pi }{e}^{-i\omega t}\tilde{{\rm{\Delta }}}{(\omega )}^{2}\cdot \\  &  & \cdot |F({k}_{\omega };\omega {)|}^{2}\frac{1}{2\omega }\frac{\partial {k}_{\omega }}{\partial \omega }\,\sum _{i,j(\ne i)}\,{e}^{i({k}_{\omega }-{k}_{0})({x}_{j}-{x}_{i})}\end{array}$$

For *ω* lies outside the energy gap, the corresponding *k*_*ω*_ is real, and we have

21 $$\sum _{j\ne i}\,{e}^{i({k}_{\omega }-{k}_{0})({x}_{j}-{x}_{i})}+1=\sum _{j}\,{e}^{i({k}_{\omega }-{k}_{0})({x}_{j}-{x}_{i})}=N\,\sum _{n}\,{\delta }_{({k}_{\omega }-{k}_{0}),\frac{2n\pi }{a}}$$

22 $$\leftrightarrow \sum _{n}\,\frac{2\pi }{a}\delta ({k}_{\omega }-{k}_{0}-\frac{2n\pi }{a})=N\,\sum _{\alpha =\langle ,\rangle }\,\frac{2\pi /a}{{|\frac{\partial {k}_{\omega }}{\partial \omega }|}_{{\omega }_{\alpha }}}\delta (\omega -{\omega }_{\alpha }({k}_{0})),$$

23 $${\rm{with}}\,{k}_{{\omega }_{\alpha }}={k}_{0},\,{\rm{and}}\,{\omega }_{ < }({k}_{0}) < \nu ,{\omega }_{ > }({k}_{0}) > \nu ,$$

here, the number of allowed lattice vectors $\frac{2\pi n}{a}$’s is the total number of site in the reciprocal space, and should be the same as that of real space, *N*.

Yet for *ω* lies within the energy gap, the corresponding *k*_*ω*_ is complex (${k}_{\omega }={k}_{\omega }^{(r)}+i\,{k}_{\omega }^{(i)}$), and we have

24 $$\begin{array}{rcl}S({k}_{\omega },{k}_{0}) & \equiv  & \sum _{n\ne 0}\,{e}^{i({k}_{\omega }-{k}_{0})na}=\sum _{n=1}^{\infty }\,({e}^{i({k}_{\omega }^{(r)}-{k}_{0})na}+{e}^{-i({k}_{\omega }^{(r)}-{k}_{0})na})\,{e}^{-{k}_{\omega }^{(i)}na}\\  & = & \frac{1}{{e}^{{k}_{\omega }^{(i)}a}{e}^{-i({k}_{\omega }^{(r)}-{k}_{0})a}-1}+\frac{1}{{e}^{{k}_{\omega }^{(i)}a}{e}^{i({k}_{\omega }^{(r)}-{k}_{0})a}-1}.\end{array}$$

Therefore, by Eqs (19–24), we have,

25 $$\begin{array}{l}{}_{{k}_{0}}\langle +|{\mathscr{U}}(t)|+\rangle _{{k}_{0}}\\ \begin{array}{ll}\quad = & \int \,\frac{d\omega }{2\pi }{e}^{-i\omega t}\tilde{{\rm{\Delta }}}(\omega )+{g}^{2}N\{\sum _{\alpha =\langle ,\rangle }\,{e}^{-i{\omega }_{\alpha }t}\tilde{{\rm{\Delta }}}{({\omega }_{\alpha }({k}_{0}))}^{2}\frac{|F({k}_{0};{\omega }_{\alpha }({k}_{0}{))|}^{2}}{2{\omega }_{\alpha }({k}_{0})\,a}\\  & +{\int }_{gap}\frac{d\omega }{2\pi }{e}^{-i\omega t}\tilde{{\rm{\Delta }}}{(\omega )}^{2}\frac{|F({k}_{\omega };\omega {)|}^{2}}{2\omega }\frac{\partial {k}_{\omega }}{\partial \omega }\frac{S({k}_{\omega },{k}_{0})}{N}\}\\  & -{g}^{2}\,\int \,\frac{d\omega }{2\pi }{e}^{-i\omega t}\tilde{{\rm{\Delta }}}{(\omega )}^{2}\frac{|F({k}_{\omega };\omega {)|}^{2}}{2\omega }\frac{\partial {k}_{\omega }}{\partial \omega }.\end{array}\end{array}$$

The last term on the RHS is much smaller than the previous ones (${\mathscr{O}}[{N}^{-1}]$), and can be ignored.

### Spontaneous emissions before renormalization

From Fig. 4(c), and by Eqs (8) and (21), the amplitude of finding a photon at site *j* at time *t* from a Dicke state initially is

26 $$\begin{array}{l}{\langle \gamma ({x}_{j})|{\mathscr{U}}(t)|+\rangle }_{{k}_{0}}\\ \begin{array}{ll}\quad = & \frac{1}{\sqrt{N}}\,\sum _{i}\,{e}^{i{k}_{0}{x}_{i}}\,\int \,dt^{\prime} \langle \gamma ({x}_{j})|{\mathscr{U}}(t-t^{\prime} )|\gamma ({x}_{i})\rangle \langle \gamma ({x}_{i})|{\mathscr{U}}(t^{\prime} )|i\rangle \\ \quad = & \frac{g}{\sqrt{N}}\,\sum _{i}\,\int \,\frac{d\omega }{2\pi }\,\int \,\frac{dk}{2\pi }{e}^{-i\omega t}{e}^{ik({x}_{j}-{x}_{i})}\tilde{G}(k,\omega )\tilde{{\rm{\Delta }}}(\omega )\,\end{array}\end{array}$$

27 $$\begin{array}{ll}\quad = & \frac{g\,{e}^{i{k}_{0}{x}_{j}}}{\sqrt{N}}\,\int \,\frac{d\omega }{2\pi }{e}^{-i\omega t}\frac{|F({k}_{\omega };\omega {)|}^{2}}{2\omega }\frac{\partial {k}_{\omega }}{\partial \omega }\tilde{{\rm{\Delta }}}(\omega )\,\sum _{i}\,{e}^{i({k}_{\omega }-{k}_{0})({x}_{j}-{x}_{i})}\\ \quad = & g\,{e}^{i{k}_{0}{x}_{j}}\sqrt{N}\{\sum _{\alpha =\langle ,\rangle }\,{e}^{-i{\omega }_{\alpha }t}\tilde{{\rm{\Delta }}}({\omega }_{\alpha })\frac{|F({k}_{0};{\omega }_{\alpha }{)|}^{2}}{2{\omega }_{\alpha }\,a}\\  & +{\int }_{gap}\,\frac{d\omega }{2\pi }{e}^{-i\omega t}\tilde{{\rm{\Delta }}}(\omega )\frac{|F({k}_{\omega };\omega {)|}^{2}}{2\omega }\frac{\partial {k}_{\omega }}{\partial \omega }\frac{\,S({k}_{\omega },{k}_{0})+1}{N}\}.\end{array}$$

Because of the many-body interactions in our system, it is necessary to do renormalization in our field-theoretical treatment. And it is shown in the Method section.

### Rabi oscillations after renormalization

Having done the renormalization, without causing confusion, we shall omit the superscript (*r*)’s for renormalized quantities hereafter, and $\tilde{{\rm{\Delta }}}(\omega )$ & $\tilde{G}(k,\omega )$ are the renormalized propagators of the two-level atom and photon defined in Eqs (42) and (43), respectively. Then the probability *P*_*D*→*D*_(*t*) of an initial Dicke state $|+{\rangle }_{{k}_{0}}$ remaining unchanged after some time evolution *t* can be rewritten from Eqs (19–25) in terms of the renormalized quantities and renormalization parameters as,

28 $${P}_{D\to D}(t)=|{}_{{k}_{0}}\langle +|{\mathscr{U}}(t)|+\rangle _{{k}_{0}}/{}_{{k}_{0}}\langle +|{\mathscr{U}}\mathrm{(0)|}+\rangle _{{k}_{0}}{|}^{2},$$

29 $$\begin{array}{l}{\rm{with}}\,\,{}_{{k}_{0}}\langle +|{\mathscr{U}}(t)|+\rangle _{{k}_{0}}\\ \begin{array}{ll}\quad = & \int \,\frac{d\omega }{2\pi }{e}^{-i\omega t}\tilde{{\rm{\Delta }}}(\omega )+{Z}_{\sigma }^{2}{Z}_{A}\,{g}^{2}N\{\sum _{\alpha =\langle ,\rangle }\,{e}^{-i{\omega }_{\alpha }t}\tilde{{\rm{\Delta }}}{({\omega }_{\alpha })}^{2}\mathrm{.}\cdot \frac{|F({k}_{0};{\omega }_{\alpha }{)|}^{2}}{2{\omega }_{\alpha }\,a}\\  & +{\int }_{gap}\,\frac{d\omega }{2\pi }{e}^{-i\omega t}\tilde{{\rm{\Delta }}}{(\omega )}^{2}\frac{|F({k}_{\omega };\omega {)|}^{2}}{2\omega }\frac{\partial {k}_{\omega }}{\partial \omega }\frac{\,S({k}_{\omega },{k}_{0})}{N}\},\end{array}\end{array}$$

and ${({}_{{k}_{0}}\langle +|{\mathscr{U}}\mathrm{(0)|}+\rangle _{{k}_{0}})}^{-\mathrm{1/2}}$ the amplitude renormalization parameter of the Dicke state $|+{\rangle }_{{k}_{0}}$.

From Fig. 4(a,b), the amplitude ${}_{{k}_{0}}\langle +|{\mathscr{U}}(t)|+\rangle _{{k}_{0}}$ is composed of two parts: the direct propagations of renormalized propagators of two-level atom, and indirect propagations from one site to another. The amplitude corresponding to direct propagations does not depend on *k*_0_. The indirect term can be further divided into two parts, the *k*_0_-wave part, and the gap part (Eq. (29)). The *k*_0_-wave part is the amplitude of the interference between two photonic Bloch waves emitted at one site and absorbed at another site; and they carry the same lattice momentum *k*_0_ but different frequencies *ω*_<_(*k*_0_) & *ω*_>_(*k*_0_). (Here we have *ω*_<_(*k*_0_) < *ν*, and *ω*_>_(*k*_0_) > *ν*). This part is much larger than the gap part (two orders of magnitude in amplitude). In addition, the closer *ω*_<_(*k*_0_) (*ω*_>_(*k*_0_)) is to the edges of the energy gap, the larger its corresponding amplitude would be. It is because that DOS of the photonic Bloch wave *ρ*_*E*_(*ω*) is very large around the energy gap (Fig. 3). For very small (large) *k*_0_, there is no corresponding *ω*_<_(*k*_0_) (*ω*_>_(*k*_0_)), and this can be seen from the photonic dispersion relation (Fig. 2). The behaviors of the evolution probabilities are results of the sum of amplitudes represented by the direct propagation and the two *k*_0_-waves.

**Figure 5:**
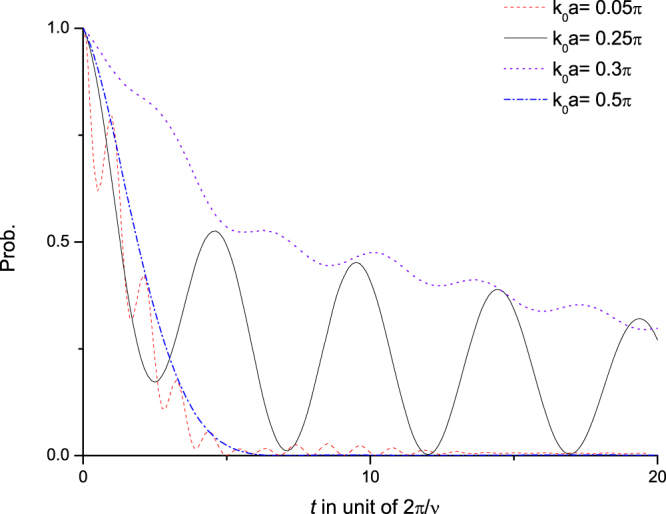
Evolution. Renormalized probabilities of Dicke states $|+{\rangle }_{{k}_{0}}$’s remaining unchanged: *k*_0_*a* = 0.05*π* (dashed), 0.25*π* (solid), 0.3*π* (dotted), 0.5*π* (dash-dotted). (Please be noted that *k*_*ν*_*a* = 0.25*π*). Here, we use the same parameters as in Fig. 2. The *x*-axis is in unit of 2*π*/*ν*, the period at the resonance angular frequency *ν*. (In the natural units (*ħ* = *c* = 1) taken in this paper, the resonance energy is *ν*. And actually it is *ħν*, if we put *ħ* back).

It can be seen from Fig. 5 that the probability *P*_*D*→*D*_(*t*) for the Dicke state $|+{\rangle }_{{k}_{0}}$ remaining unchanged shows quite different behaviors for different *k*_0_’s (the momentum at resonance *k*(*ν*) = 0.25*π*/*a*):

- For very small *k*_0_ (the curve *k*_0_*a* = 0.05*π* in Fig. 5), there is only one *k*_0_-wave with energy ${\omega }_{ < }({k}_{0})\ll \nu$. In this case, DOS *ρ*_*E*_(*ω*_<_) is small, but the factor 1/*ω*_<_ in Eq. (29) becomes very large. Therefore, the amplitude corresponding to the *k*_0_-wave with energy *ω*_<_(*k*_0_) is not too small compared with that at ${k}_{0}\sim k(\nu )$. But the amplitude corresponding to the direct propagation is very small compared to that of the indirect propagation. Their interference shows small wiggling.
- For ${k}_{0}\sim k(\nu )$ (the curve *k*_0_*a* = 0.25*π* in Fig. 5), two *k*_0_-waves exist, and their energies are close to the edges of the energy gap. Therefore, the amplitudes of both *k*_0_-waves are large because of their high densities of state around the energy gap. As a result, the interference of the two *k*_0_-waves is notable, and the evolution probability *P*_*D*→*D*_(*t*) is significantly oscillatory.
- For *k*_0_ which is not much away from *k*(*ν*) (the curve *k*_0_*a* = 0.3*π* in Fig. 5), two *k*_0_-waves exist and *ω*_<_(*k*_0_) is close to the energy gap, but *ω*_>_(*k*_0_) is not. Therefore, the amplitude of the *k*_0_-wave corresponding to *ω*_>_(*k*_0_) is small because of its small DOS. Thus, the interference between the two *k*_0_-waves is small, and *P*_*D*→*D*_(*t*) is far less oscillatory.
- For *k*_0_ which is much larger than *k*(*ν*) (the curve *k*_0_*a* = 0.5*π* in Fig. 5), there is only one *k*_0_-wave with energy ${\omega }_{ > }({k}_{0})\sim 2\nu$. And its amplitude is very small because both the factor 1/*ω*_<_ & DOS are small. As a result, only the amplitude corresponding to direct propagation is significant; and there is almost no interference at all.

We can see that, for different incident momenta *k*_0_’s, the Rabi oscillations show very different oscillatory behaviors in decay. If it were not for the nonlinear photonic dispersion relation with energy gap and the non-Lorentzian DOS of photonic Bloch wave, the interference performances shown in the behaviors of *P*_*D*→*D*_(*t*) of Rabi oscillations would not be so rich.

### Spontaneous emissions after renormalization

Similarly, the probability ${P}_{D\to \gamma ({x}_{j})}(t)$ of finding a photon at site *j* after some time evolution *t* from a Dicke state $|+{\rangle }_{{k}_{0}}$ initially (Fig. 4(c)) can be rewritten from Eqs (26) and (27) in terms of the renormalized quantities and renormalization parameters as,

30 $${P}_{D\to \gamma ({x}_{j})}(t)={|{\langle \gamma ({x}_{j})|{\mathscr{U}}(t)|+\rangle }_{{k}_{0}}/\sqrt{{}_{{k}_{0}}\langle +|{\mathscr{U}}\mathrm{(0)|}+\rangle _{{k}_{0}}}|}^{2},$$

31 $$\begin{array}{l}{\rm{with}}\,{\langle \gamma ({x}_{j})|{\mathscr{U}}(t)|+\rangle }_{{k}_{0}}\\ \begin{array}{ll}\quad = & {Z}_{\sigma }^{2}\,{Z}_{A}\,g\,{e}^{i{k}_{0}{x}_{j}}\sqrt{N}\{\sum _{\alpha =\langle ,\rangle }\,{e}^{-i{\omega }_{\alpha }t}\tilde{{\rm{\Delta }}}({\omega }_{\alpha })\frac{|F({k}_{0};{\omega }_{\alpha }{)|}^{2}}{2{\omega }_{\alpha }\,a}\\  & +{\int }_{gap}\,\frac{d\omega }{2\pi }{e}^{-i\omega t}\tilde{{\rm{\Delta }}}(\omega )\frac{|F({k}_{\omega };\omega {)|}^{2}}{2\omega }\frac{\partial {k}_{\omega }}{\partial \omega }\frac{\,S({k}_{\omega },{k}_{0})+1}{N}\}.\end{array}\end{array}$$

As is in Eq. (29), the amplitude ${\langle \gamma ({x}_{j})|{\mathscr{U}}(t)|+\rangle }_{{k}_{0}}$ (Eq. (31)) is composed of two parts: the *k*_0_-wave part and the gap part; but they are different from their counterparts in Eq. (29) with the power of the renormalized propagator of the two-level atom $\tilde{{\rm{\Delta }}}(\omega )$ in the integrand to be 1 rather than 2. The *k*_0_-wave part here is also the interference of two light waves of the same lattice momentum *k*_0_ but with different frequencies *ω*_<_(*k*_0_) & *ω*_>_(*k*_0_). This part is even larger than the gap part (three orders of magnitude in amplitude).

**Figure 6:**
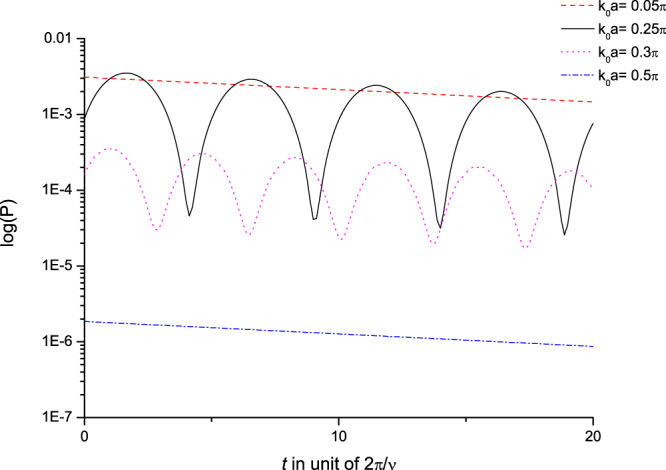
Emission. Renormalized probabilities of spontaneous emission of Dicke states $|+{\rangle }_{{k}_{0}}$’s in log scale: *k*_0_*a* = 0.05*π* (dashed), 0.25*π* (solid), 0.3*π* (dotted), 0.5*π* (dash-dotted). (Please be noted that *k*_*ν*_*a* = 0.25*π*). The *x*-axis is in unit of 2*π*/*ν*, the period at the resonance angular frequency *ν*. Here, we use the same parameters as in Fig. 2.

From Fig. 6, the spontaneous emission of a Dicke state ${P}_{D\to \gamma ({x}_{j})}(t)$ shows significantly different behaviors for different *k*_0_’s. Most of them can be understood in a similar way from explanations we made with *k*_0_-waves and the density of photonic Bloch state in the previous subsection except that there is no direct term here (Eq. (31). In summary, we have two things to point out:

- It shows strong radiance for ${k}_{0}\sim 0$ & ${k}_{0}\sim k(\nu )$, but getting weaker notably for *k*_0_ is away from 0 & *k*(*ν*). The difference could go up to 5 orders of magnitude. That is, besides the superradiance occurring at long wavelengths (∝1/*ω*_<_(*k*_0_) see Eq. (31)), the spontaneous emission is also very strong near the energy gap because of the high DOS in this energy range.
- It shows significant oscillatory behavior as ${k}_{0}\sim k(\nu )$; but it is far less oscillatory as *k*_0_ is away from *k*(*ν*). Therefore, for different incident momenta *k*_0_’s, the spontaneous emissions show very different behaviors in magnitudes and oscillations.

We studied the system of an arrayed two-level atoms (with energy spacing *ν*) interacting with a photonic field via a quantum interaction. We take into account the multi-scatterings between photon and *N* two-level atoms in our calculations. For ${g}^{2}\sim \nu \mathrm{/100}$, the obtained photonic dispersion relation is almost linear but with a peak followed by a dip as the photonic energy is around the energy spacing *ν*. Within such a range of photonic energy ($\omega \sim \nu$), the corresponding lattice momentum *k*_*ω*_ is complex and the photon is in the attenuated mode. In fact, this agrees with our physical intuitions that photon will be absorbed as its energy is around the spacing between two atomic energy levels. Thus, there is an energy gap in the photonic dispersion relation. And for photon propagating in a lattice of two-level atoms, its eigenfunction ought to be Bloch wave rather than plane wave. Consequently, the propagator of the dressed photon is modified significantly. In addition, due to repeated atom-light interactions, there is also a dynamical correction to the atomic energy spacing *ν* → *ν*(*ω*) = *ν* + *δν*(*ω*). Accordingly, as the photonic propagator, the propagator of the two-level atom is modified notably, too.

In a field-theoretical treatment like ours, we need to go through the renormalization process to calculate physical results. The renormalization scheme is that we first adopt appropriate renormalization conditions, and from them we solve the renormalization parameters. Afterwards, we present our results with the renormalized quantities. There follows several interesting results which are distinct from those obtained through a linear dispersion relation of free photon. For example, DOS of dressed photon is non-Lorentzian and is very large near the energy gap around which slow light can exist; the Rabi oscillations become monotonically decreasing in some cases; and besides the super-radiance occurs at long wavelengths, the spontaneous emission is also very strong near the energy gap because of the high DOS.

There are systems that mediate interactions between one-dimensional fields and two-level-system array such as an atom array coupled to photonic fields^23–26,29,30,45,46^, a superconducting qubit array coupled to transmission-line resonator^47–50^, a gate-control dot array coupled to microwave photons^51^. And they can be realized in experiments. In these systems, our results can be applied to the studies of slow (storage) light and the quantum memory in the atomic medium^31^, the optical nonlinearity^52–54^ and the multipartite quantum entanglement^55,56^.

## Methods

### Renormalization

The scheme of the renormalization process is that we first treat the original field operators & coupling constant as the bare quantities which are the products of the renormalized quantities and the renormalization parameters,

32 $${\sigma }^{(b)}=\sqrt{{Z}_{\sigma }}\,{\sigma }^{(r)},$$

33 $${A}^{(b)}=\sqrt{{Z}_{A}}\,{A}^{(r)},$$

34 $${g}^{(b)}={Z}_{g}\,{g}^{(r)}.$$

And then the renormalization parameters (*Z*’s) are determined through appropriate renormalization conditions which are adopted as

35 $$\langle i|{\mathscr{U}}(t=\mathrm{0)|}i\rangle =1,$$

36 $$\langle \gamma ({x}_{i})|{\mathscr{U}}(t=\mathrm{0)|}\gamma ({x}_{i})\rangle =1,$$

37 $${g}^{(b)}{A}^{(b)\dagger }\,{\sigma }^{(b)}={g}^{(r)}{A}^{(r)\dagger }\,{\sigma }^{(r)}.$$

The first two conditions are that the states of the two-level atom and the photon will keep unchanged as no time evolution is involved. The third condition is due to the fact that our model is bilinear and there is no renormalization correction to the physical quantities from loop diagrams. Thus, the renormalization of the coupling constant is only from amplitude renormalizations of the field operators in the interaction term of the effective potential^57^ Γ derived from the partition function of the action, and is formally the same as the interaction term of the Hamiltonian (Eq. (37)).

By the above renormalization conditions, Eqs (35–37), we have,

38 $$\begin{array}{rcl}1 & = & \langle i|{\mathscr{U}}\mathrm{(0)|}i\rangle =\langle T{\sigma }^{(r)\dagger }{\mathrm{(0}}^{+}){\sigma }^{(r)}\mathrm{(0)}\rangle =\int \,\frac{d\omega }{2\pi }{\tilde{{\rm{\Delta }}}}^{(r)}(\omega )\\  & = & {Z}_{\sigma }^{-1}\langle T{\sigma }^{(b)\dagger }{\mathrm{(0}}^{+}){\sigma }^{(b)}\mathrm{(0)}\rangle ={Z}_{\sigma }^{-1}\,\int \,\frac{d\omega }{2\pi }\tilde{{\rm{\Delta }}}(\omega ),\end{array}$$

39 $$\begin{array}{rcl}1 & = & \langle \gamma ({x}_{i})|{\mathscr{U}}\mathrm{(0)|}\gamma ({x}_{i})\rangle =\langle T\,{A}^{(r)\dagger }{\mathrm{(0}}^{+}){A}^{(r)}\mathrm{(0)}\rangle =\int \,\frac{d\omega }{2\pi }\frac{dk}{2\pi }{\tilde{G}}^{(r)}(k,\omega )\end{array}$$

40 $$\begin{array}{rcl} & = & {Z}_{A}^{-1}\langle T\,{A}^{(b)\dagger }{\mathrm{(0}}^{+}){A}^{(b)}\mathrm{(0)}\rangle ={Z}_{A}^{-1}\,\int \,\frac{d\omega }{2\pi }\frac{dk}{2\pi }\tilde{G}(k,\omega ),\end{array}$$

41 $$\begin{array}{rcl}{\rm{and}}\,{Z}_{g} & = & {Z}_{\sigma }^{-\mathrm{1/2}}\,{Z}_{A}^{-\mathrm{1/2}},\end{array}$$

where $\tilde{{\rm{\Delta }}}(\omega )$ & $\tilde{G}(k,\omega )$ are bare propagators listed in Eqs (8) and (15), respectively. Please notice that the *g* appearing in the above two equations (explicitly in Eq. (15), and implicitly in |*F*|^2^ in both Eqs (8) and (15) is ${g}^{(b)}={Z}_{\sigma }^{-\mathrm{1/2}}\,{Z}_{A}^{-\mathrm{1/2}}\,{g}^{(r)}$ (Eq. (41)). And we have |*F*^(*b*)^| = *Z*_*σ*_*Z*_*A*_|*F*^(*r*)^|. Finally, our renormalization scheme boils down to solving two unknowns (*Z*_*σ*_ & *Z*_*A*_) from two equations (Eqs (38) and (40)). Afterwards, all bare quantities will be replaced and we can then present our calculations in terms of the renormalized quantities only. For example, the renormalized propagators of the two-level atom and photon are,

42 $$i{\tilde{{\rm{\Delta }}}}^{(r)}{(\omega )}^{-1}={Z}_{\sigma }\cdot [\omega -\nu +i\delta +i{g}^{(r)2}{Z}_{\sigma }\,{Z}_{A}\frac{|{F}^{(r)}({k}_{\omega };\omega {)|}^{2}}{2\omega }(\frac{\partial {k}_{\omega }}{\partial \omega })],$$

43 $${\tilde{G}}^{(r)}(k,\omega )={Z}_{\sigma }^{2}\,{Z}_{A}\cdot |{F}^{(r)}(k;{\omega }_{k}{)|}^{2}\frac{i}{{\omega }^{2}-{\omega }_{k}^{2}+i\epsilon }.$$
